# Development and Evaluation of Oral Controlled Release Chlorpheniramine-Ion Exchange Resinate Suspension

**DOI:** 10.4103/0250-474X.44613

**Published:** 2008

**Authors:** A. U. Kadam, D. M. Sakarkar, P. S. Kawtikwar

**Affiliations:** S. N. Institute of Pharmacy, Pusad, Dist. Yavatmal-445 204, India

**Keywords:** Controlled release, chlorpheniramine maleate, ion-exchange resin, formulation, Indion 244, suspending agent

## Abstract

An oral controlled release suspension of chlorpheniramine maleate was prepared using ion-exchange resin technology. A strong cation exchange resin Indion 244 was utilized for the sorption of the drug and the drug resinates was evaluated for various physical and chemical parameters. The drug-resinate complex was microencapsulated with a polymer Eudragit RS 100 to further retard the release characteristics. Both the drug-resinate complex and microencapsulated drug resinate were suspended in a palatable aqueous suspension base and were evaluated for controlled release characteristic. Stability study indicated that elevated temperature did not alter the sustained release nature of the dosage form indicating that polymer membrane surrounding the core material remained intact throughout the storage period.

Much of the research effort in developing novel drug delivery systems has centered on sustained or controlled release of drug from dosage forms. Most of the new peroral sustained release preparations are solid dosage forms, i.e. tablets or capsules. Only recently has attention been devoted to liquid sustained release preparations which may be more palatable to pediatric patients^1^.

Chlorpheniramine is commonly used as an antihistaminic for allergic rhinitis, especially in conjunction with pseudoephedrine. The dosage interval for chlorpheniramine ranges from 4 h to 6 h, even though the elimination half life has been reported as high as 20 h^2^. A dosage form which releases chlorpherinamine slowly over a 12 h period would have substantial advantages over convential, immediate release preparations.

In the present study, Indion 244 a strong cation exchange resin containing free sulphonic acid group on cross linked polystyrene matrix was selected for sorption of chlorpherinamine^3^. The molecule of chlorpherinamine maleate has a tertiary amine group. This can be exchanged for the hydrogen present on the cation exchange resin.

The drug resinate complex was prepared and this microcapsules where formulated into suspension containing different suspending agents and the effect of aging and temperature on the drug release profile of various suspended microcapsules was determined.

Indion 244 obtained as gift sample from Ion exchange resin India Ltd. Chlorpheniramine maleate procured from Loba Chemicals and Eudragit RS 100 procured from Colorcon Ltd.

Indion 244 was given a pretreatment to remove the impurities associated with industrial scale manufacture. The resin was treated with five to ten bed volumes of 1 N NaOH solution and 1 N HCl alternatively in order to eliminate resin derived products in the solutions^4^. Demineralised water was used to prepare all the solutions. The resin in hydrogen form was evaluated for moisture content, particle size and cation exchange capacity.

Preparation of drug-resinate was tried by batch method, as efficient elution of drug solution was not possible from the column due to its small particle size and hence batch method was preferred. Accurately weighted chlorpherinamine was dissolved in 200 ml demineralised water and Indion 244 was added, the quantity taken was in 1:1 proportion. This mixture was stirred for 4 h and filtered through Whatman filter paper. The drug-resinate was washed with demineralised water till the washing showed no traces of the drug^5^. Drug loading was determined spectrophotometrically. fig. 1 shows the time required for maximum drug loading. The drug-resinate was evaluated for drug content and *in vitro* drug release.

**Fig. 1:**
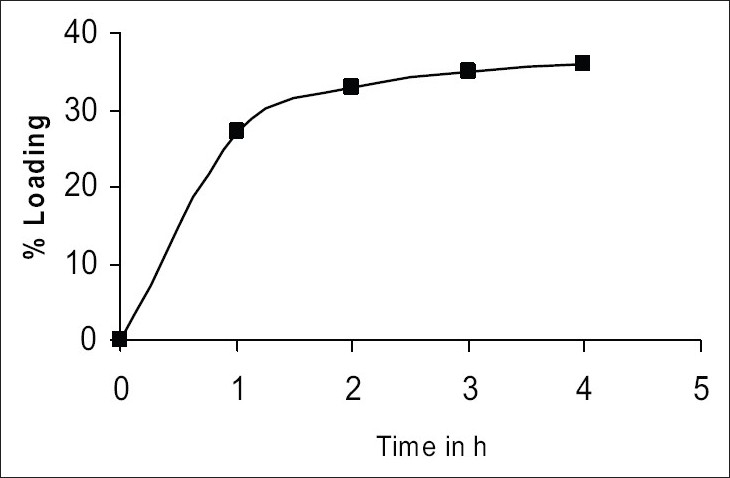
Sorption of chlorpherinamine maleate on Indion 244

Accurately weighted 0.1 g of drug-resinate was stirred with 250 ml of 0.1 M CaCl_2_for 2 h^6^. This was filtered and after suitable dilution drug content was estimated spectrophometrically at 262 nm. The drug-resinate was weighed accurately and placed in USP rotating basket method employing 900 ml of dissolution medium pH 1.2 for first 2 h and 7.2 pH for remaining 6 h maintained at 37±0.5°. The speed of the basket was maintained at 50 rpm. The release pattern of drug-resinate are shown in fig. 2. To further retard the drug release, the resinate particles were coated with Eudragit RS 100 (5 – 20% w/w). Microencapsulation was carried out by solvent evaporation technique^6^.

**Fig. 2:**
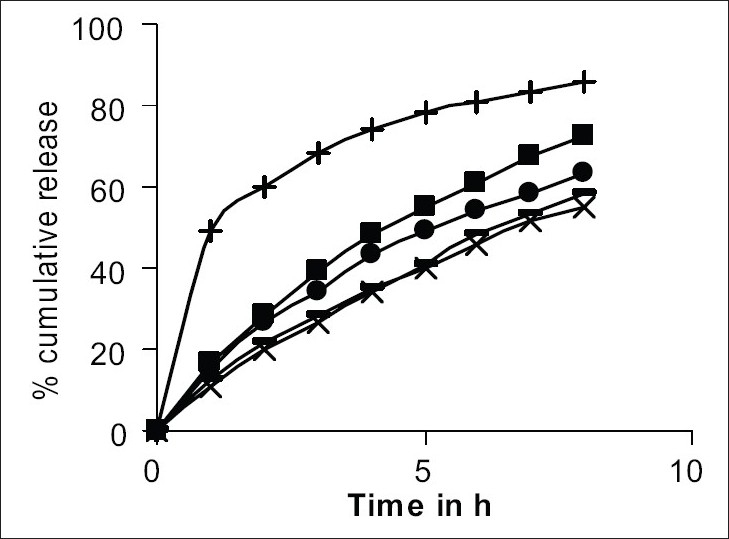
*In vitro* release profile of drug-resinate and microencapsulated drug-resinate. *In vitro* release profile of drug-resinate and microencapsulated drugresinate with Eudragit RS 100. (5-20% w/w). (—+— )% Cumulative drug release profile of drug resinate, (—■—)-microencapsulated with 5% Eudragit RS 100, (—●—) Eudragit RS 100 10%, (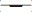) Eudragit RS 100 15% and (—×—) Eudragit RS 100 20%.

The drug-resinate was stirred with polymer solution for 2 h. The solvent was evaporated by continuous stirring on a water bath. Microencapsulated product was dried at 40° and evaluated for drug content, content uniformity, percent coating and *in vitro* drug release. The results are given in Table 1. The *in vitro* drug release from drug-resinate was significantly retarded after coating as shown in fig. 2. Microcapsules obtained by coating the drug-resinate with 10% Eudragit RS 100 were selected for the formulation of suspension.

**TABLE 1:** EVALUATION OF MICROENCAPSULATED DRUG - RESINATE

Test	5% Eudragit	10% Eudragit	15% Eudragit	20% Eudragit
Nature	Free flowing	Free flowing	Free flowing	Cohesive, Lumpy
Drug content (%w/w)	34.01	33.0	32.49	31.67
Content uniformity (%w/w)	33.99 - 34.02	32.99 - 33.01	32.48 - 32.49	31.65 - 31.68
Coating (%w/w)	4.95	9.98	14.97	19.99
Particle size (μm)	62-142	72-158	85-167	93-177
% *in vitro* drug release in 8 h	72	63	58	55

Microcapsules were formulated into aqueous suspension containing different suspending agent's viz. Gaur gum (0.8-1.0), methylcellulose 4000 cps (1.0-1.5), Xanthan gum (0.4-0.6) was tried^7^. All formulation was evaluated for physicochemical parameters. Xanthan gum formulation having minimum sedimentation rate and excellent redispersibility were found to be better. The optimum formulation contained xanthan gum (0.4% w/v), sorbital (40% v/v), sucrose (40% w/v), Tween 20 (0.1% v/v), and sodium methylparaben in a coloured and flavored aqueous base. The suspensions of drug-resinates and microcapsules were prepared and their physicochemical parameters were compared. Compatibility of the ingredients with coated and uncoated drug resinate was also checked.

The suspension was evaluated for aesthetic appeal, pH, particle size analysis, wt/ml, sedimentation rate, redispersibility, viscosity, drug content and *in vitro* drug release pattern^8^. The results are given in Table 2. Leaching of the drug from the resinate complex into the aqueous suspension vehicle was also studied.

**TABLE 2:** EVALUATION OF SUSPENSION CONTAINING MICROENCAPSULATED DRUG-RESINATE (MDR) AND UNCOATED DRUG- RESINATE COMPLEX (UDR)

Test	Observation MDR	Observation UDR
Appearance	uniform	uniform
Taste	sweet palatable	sweet palatable
pH	6.01	5.75
Viscosity (cps)	390	360
Sedimentation	0.96	0.92
Particle size	74 - 160	45 - 140
Wt per ml	1.42	1.22
Redispersibility	+++	+++
Drug content	100.07	99.24
Drug eluted in the vehicle	0.13	0.44
% *In vitro* release	62.85	86.15

Finally the formulation containing coated drug-resinate were subjected to accelerated stability studies where the representative samples were stored at various temperatures viz. room temperature, 37°, 45°. After giving pretreatment to resin, the resin in hydrogen form was evaluated for moisture content, particle size and cation exchange capacity and they were found to be 3.7% w/w, 31-145 μm and 5.49 mEq\g dry resin, respectively.

The process for preparing drug resinate was optimized with respect to methodology, drug resin proportion and time for sorption. Loading was tried by batch method, as efficient elution of the drug from column was not possible. The drug resin proportion of 1:1 achieved equilibrium in 4 h as shown in fig. 1 and 36% w/w of drug loading was possible by this method. These drugs resinate released 86% of the drug in 8 h as shown in fig. 2.

The uncoated chlorpheniramine resin complex released the drug rapidly. To further retard the release drug-resinate complex was coated with Eudragit RS-100 (5-20%w/w). In the coated drug resinate the drug release was found to decrease from 72% to 55% in 8 h as shown in fig. 2. The drug resinate microcapsules coated with 10% was selected for formulation of suspension, as only 63% of drug was released in 8 h.

The plain drug resinate and microcapsules coated with 10% Eudragit RS-100 were formulated separately into suspension, the optimum suspension formulation then prepared using 0.4% Xanthan gum was evaluated for physiochemical parameters as shown in Table 2. Drug leached in the vehicle at room temperature for coated drug resinate was 0.13%w/w and plain drug resinate was 0.44% w/w indicating better protection offered by Eudragit RS-100. Suspension containing 0.4% Xanthan gum was selected for extended stability studies.

The suspension stability studies showed that microcapsule could be suspended in to suspension without seriously compromising the sustained release properties of the dosage form. It was also seen that for various storage temperature conditions there was minimal drug leaching into the suspension from drug-loaded microspheres. This coupled with very negligible change in the pH and viscosity, indicating better physiochemical stability of the formulated suspension (Table 3). These results indicate that the polymer membrane surrounding the core material remained intact throughout the storage period. The formulation containing microcapsules was found to be stable. Thus drug resinate complex has proved to be an efficient carrier for controlled release oral liquid suspension of chlorpheniramine maleate.

**TABLE 3:** EFFECT OF AGING AT ELEVATED TEMPERATURES FROM SUSPENSED MICROCAPSULE

Days	Temp	Drug Content (%)	Viscosity (cps)	% Drug Leaching	pH	% Cumulative Drug Release
1	Room temp.	100.4	390	0	6.12	63.010
	37^°^	100.4	390	0	6.12	63.010
	45^°^	100.4	390	0	6.12	63.010
7	Room temp.	99.50	385	0.15	6.12	62.099
	37^°^	99.50	381	0.19	6.06	62.011
	45^°^	98.80	377	0.22	6.02	61.021
14	Room temp.	98.16	383	0.18	5.79	62.071
	37^°^	97.85	379	0.23	5.55	61.991
	45^°^	97.00	374	0.29	5.85	60.010
21	Room temp.	97.30	381	0.24	5.62	61.104
	37^°^	96.45	376	0.28	5.46	60.112
	45^°^	96.02	372	0.36	5.43	59.110
30	Room temp.	97.00	380	0.30	5.36	60.811
	37^°^	96.25	374	0.38	5.29	58.079
	45^°^	95.86	370	0.45	5.26	56.071
